# Supplementary material for: Regulating TiO2 Deposition Using a Single-Anchored Ligand for High-Efficiency Perovskite Solar Cells
Source: Materials (Basel). 2024 Aug 2;17(15):3820. doi: 10.3390/ma17153820 (PMC11312953; doi:10.3390/ma17153820)
Supplement: Supplementary file 1 [file materials-17-03820-s001.zip › materials-3060667-supplementary.pdf]

materials

Supplementary Materials

Regulating TiO2 deposition using single-anchored ligand for high-efficiency perovskite solar cells

Zhanpeng Xu [1], Zhineng Lan [2], Fuxin Chen [1], Chon Yin [1], Longze Wang [2], Zhehan Li [2], Luyao Yan [2] and Jun Ji [3,*]

[1]Power China Huadong Engineering Corporation Limited Hangzhou, 311122, China
[2]State Key Laboratory of Alternate Electrical Power System with Renewable Energy Sources, North China Electric Power University, Beijing 102206, China
[3]Beijing Huairou Laboratory, Beijing 101400, China
* Correspondence: author: jijun@hrl.ac.cn

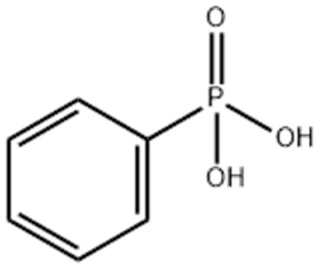

Figure S1. The molecular structure formula of phenylphosphonic acid.

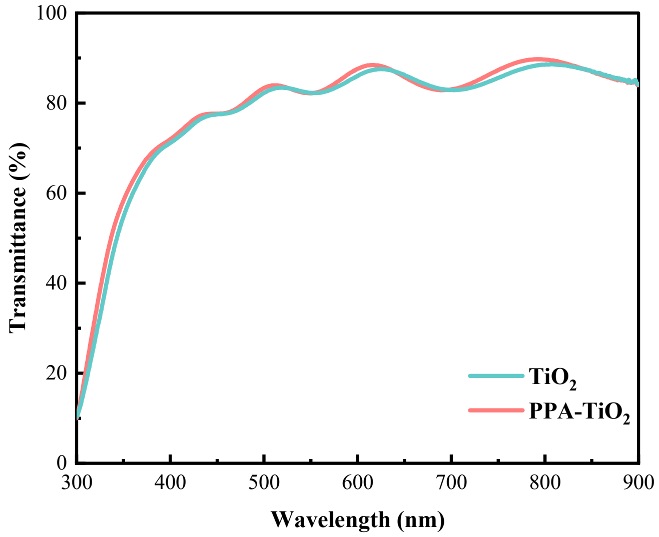

Figure S2. Optical transmittance spectra of ETLs.

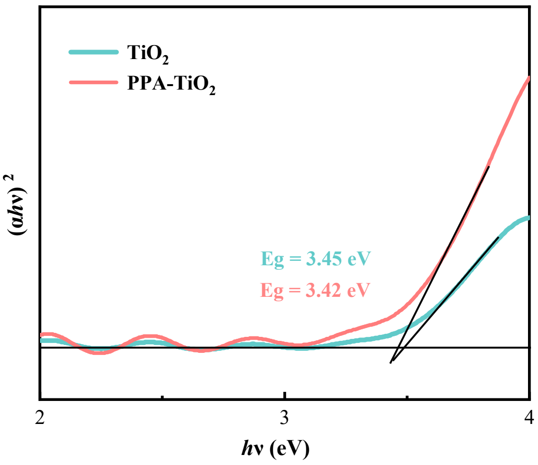

Figure S3. Tauc plots of ETLs.

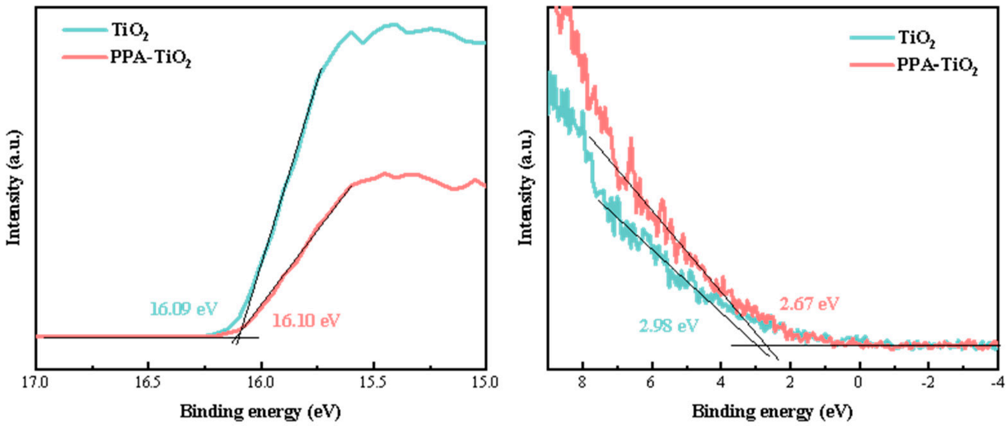

Figure S4. UPS profiles of ETLs.

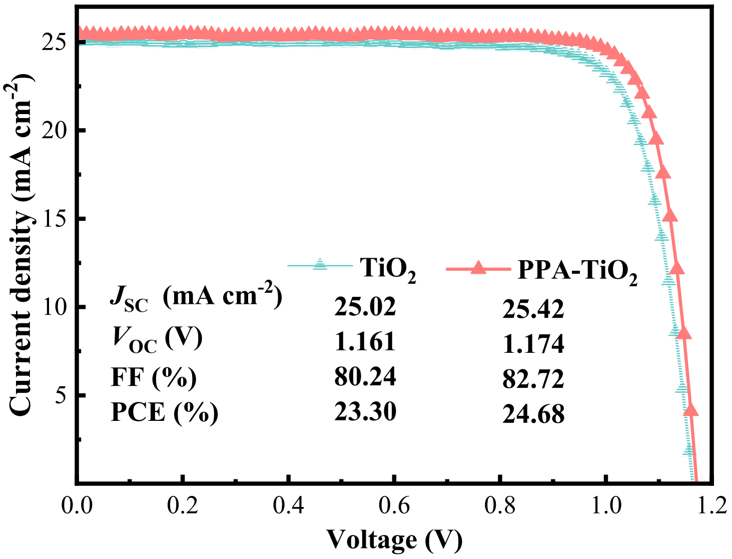

Figure S5. Forward scan *J*–*V* curves of PSCs with different ETLs.

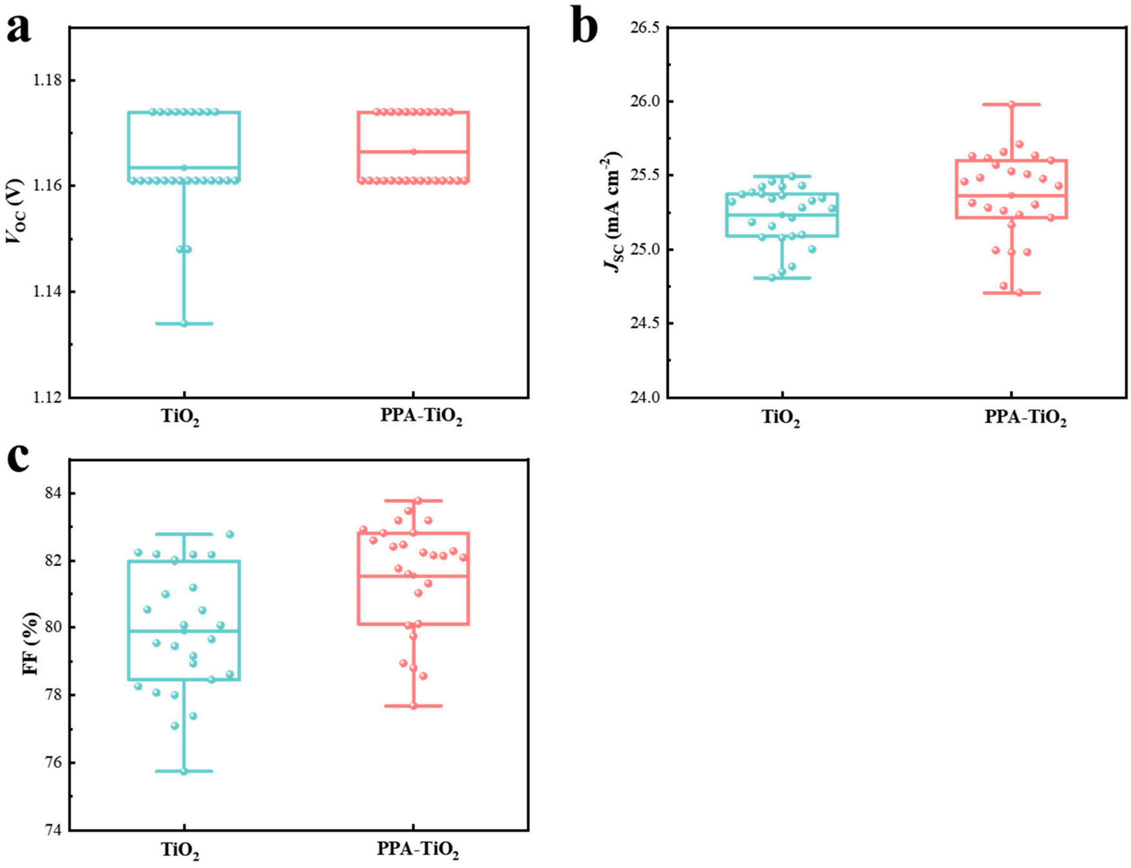

Figure S6. Distribution of the photovoltaic parameters for PSCs based on different ETLs. (a) distribution of V_{OC}; (b) distribution of J_{SC}; (c) distribution of FF.

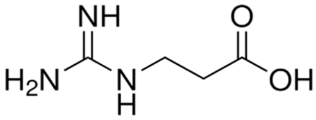

Figure S7. The molecular formula of the β-Guanidinopropionic acid.

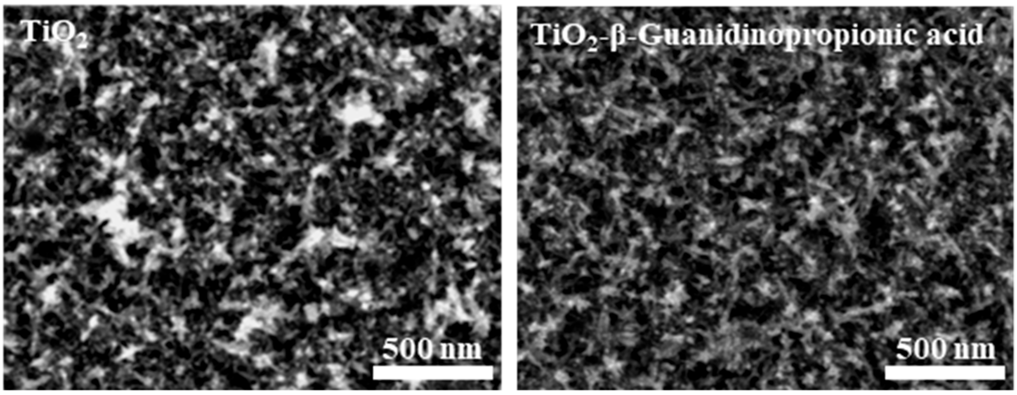

Figure S8. Surface SEM images of ETLs, respectively.

Note S1.To demonstrate the universality of the single-anchored ligand regulation strategy, we used β-Guanidinopropionic acid (Figure S2), which contains a carboxyl

group that can bind to TiO_2, and the molecular skeleton and the guanidine group that acts as the steric hindrance, to regulate the CBD process of TiO_2. As predicted, many dendrite-like aggregates appear on the surface of the normal TiO_2 ETL, which makes the surface very rough. However, the surface of the ETL film with β-Guanidinopropionic acid regulation is smoother, and there is no obvious agglomeration (Figure S3).
